# Improved flux profiling in genome-scale modeling of human cell metabolism

**DOI:** 10.1016/j.crmeth.2025.101275

**Published:** 2026-01-12

**Authors:** Cyriel A.M. Huijer, Xiang Jiao, Yun Chen, Rosemary Yu

**Affiliations:** 1 Department of Molecular Developmental Biology, Radboud Institute for Molecular Life Sciences, Faculty of Science, Radboud University, 6525GA Nijmegen, the Netherlands; 2 Department of Life Sciences, Chalmers University of Technology, 412 96 Gothenburg, Sweden

**Keywords:** cell metabolism, genome-scale metabolic modeling, flux profiling, feasible flux space

## Abstract

Understanding human cell metabolism through genome-scale flux profiling is of interest to diverse research areas of human health and disease. Metabolic modeling using genome-scale metabolic models (GEMs) has the potential to achieve this, but has been limited by a lack of appropriate input data as model constraints. Here, we compare the commonly used consumption and release (CORE) method to a regression-based method (regression during exponential growth phase; REGP). We found that the CORE method is not reliable despite being prevalent in human studies, whereas the exchange fluxes determined by REGP provide constraints that substantially improve GEM simulations for human cell lines. Our results show that the GEM-simulated feasible flux space is constrained to a biologically plausible region, allowing an exploration of the basic organizing principles of the feasible flux space. These improvements help to fulfill the promise of GEMs as a valuable tool in the study of human metabolism and future development of translational applications.

## Highlights

• Regression-based method satisfies the steady-state assumption required for FBA/FVA
• REGP- and CORE-calculated exchange fluxes differ substantially
• REGP-calculated fluxes constrain GEM models to biologically plausible solutions

## Motivation

As cellular metabolism plays a central role in human health and disease, genome-scale flux profiling is of interest to a wide range of research fields. Genome-scale metabolic modeling is a promising computational tool that can be used to simulate intracellular metabolic fluxes in human cells. However, these models are known to be sensitive to the quality of the constraints, which are often calculated based on single-time point data that violate steady-state assumptions. In this paper, we investigate the potential of a regression-based method using multi-time point data to enable more accurate metabolic simulations.

## Graphical abstract

**Figure:**
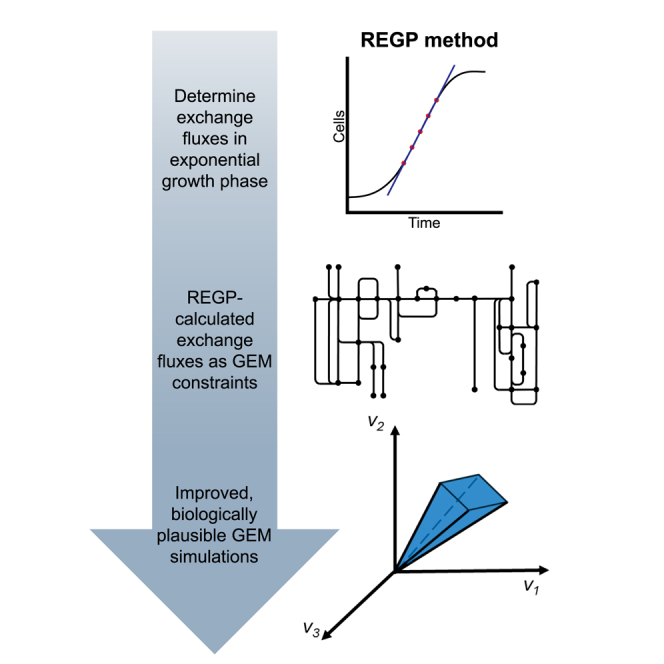

## Teaser

Huijer et al. present a regression-based method to accurately determine metabolic exchange fluxes in human cells. These fluxes provide reliable constraints for metabolic model simulations to study human metabolism and disease.

## Introduction

Metabolism of human cells is a highly complex network of thousands of metabolites and reactions. Alterations in cell metabolism are associated with many complex health conditions such as diabetes, inflammatory diseases, and cancer.^1^^,^^2^ Importantly, the defining feature of metabolism is not the concentrations of metabolites in the cell but the metabolic fluxes (*r*) through reactions and pathways.^3^^,^^4^ Intracellular metabolic fluxes can be experimentally determined through isotope-labeled substrate tracing for a small subset of reactions,^5^^,^^6^ but to systematically profile all fluxes of a cell at the genome scale, mathematical modeling is necessary.

Genome-scale metabolic model (GEM) is a modeling framework wherein the complete metabolic network of a cell is reconstructed *in silico*.^5^^,^^7^ GEMs can be used for simulations to calculate the optimal (max or min) fluxes of each reaction and determine the feasible flux space for the entire metabolic network, using techniques called flux balance analysis (FBA) and flux variability analysis (FVA).^8^ FBA and FVA require a small amount of input data as constraints, typically consisting of measured exchange fluxes (± measurement error) of a small number of exo-metabolites, such as glucose, lactate, and amino acids. With these experimentally measured input data, GEM simulations have been shown to be strikingly accurate in microorganisms such as *E. coli* and *S. cerevisiae*.^9^^,^^10^^,^^11^ On the other hand, the predictive power of GEMs can vary considerably and often depends on the quality of the constraints.^12^ This variability underscores the importance of collecting high-quality measurements to improve GEM simulation accuracy. Critically, FBA and FVA assume that cells are in pseudo-steady state. Thus, input data for these successful applications of GEM simulations have all been collected during exponential growth.

**Figure 1:**
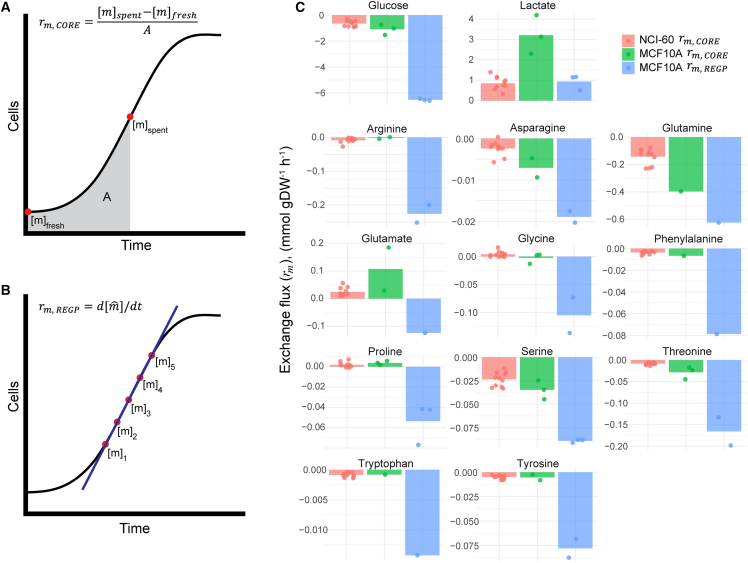
Exo-metabolite exchange fluxes (A and B) Schematic overview of exo-metabolite exchange flux calculation by the CORE and REGP methods. [*m*], exo-metabolite concentration; A, area under the curve; $\left[\hat{m}\right]$, linear-regression-fitted exo-metabolite concentration (see STAR Methods section). (C) Exo-metabolite exchange fluxes (*r*_*m*_) for 11 cell lines in the NCI-60 panel calculated by the CORE method and for the MCF10A cell line calculated by either the CORE or the REGP method. See also Table S1.

Building on the success of GEM simulations in microbial applications, there is considerable interest in studying human cell metabolism using Human-GEM.^3^^,^^13^^,^^14^ These studies determined exo-metabolite exchange fluxes in human cells by the consumption and release (CORE)^4^ method (Figure 1A). In this method, exo-metabolite concentrations in the cell culture media are measured at a single time point and exchange fluxes are then calculated based on the difference between the measured “spent” media and the unused (“fresh”) media (Figure 1A). Thus, CORE-calculated exchange fluxes are not true steady-state exchange fluxes, and the use of these values as constraints for FBA and FVA should be done with caution.

Regression-based methods for estimating exchange fluxes have been applied in microorganisms^15^ and mammalian studies.^16^^,^^17^^,^^18^^,^^19^ In this study, we adapted a regression-based approach to calculate exchange fluxes in human cells, which we refer to as REGP (regression during exponential growth phase; Figure 1B), and compared it to the conventional CORE method. We found that REGP-calculated exchange fluxes (*r*_*m*,*REGP*_) were substantially different from CORE-calculated exchange fluxes (*r*_*m*,*CORE*_) (Figure 1C). Unlike models constrained with *r*_*m*,*CORE*_, the model with *r*_*m*,*REGP*_ as input data for FBA and FVA produced feasible GEM simulations. Additionally, we showed that the GEM-simulated feasible flux space was constrained to a more biologically plausible region, allowing an exploration of the basic organizing principles of the feasible flux space. We anticipate that incorporating regression-based exchange fluxes from human cell lines as input for GEM simulations can rapidly advance our understanding of cell metabolism in diverse applications related to human health and disease.

## Results

### Exo-metabolite exchange fluxes at steady state

**Figure 2:**
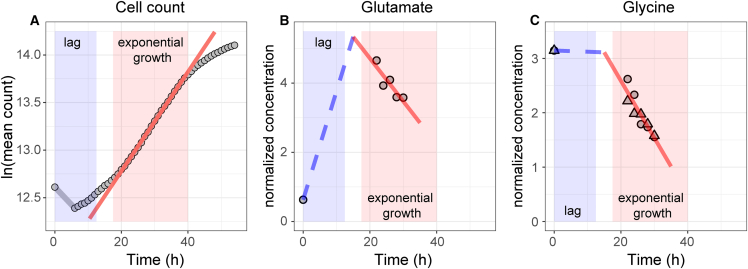
Exo-metabolite exchange fluxes in different growth phases (A) Growth curve of MCF10A cells showing a distinct lag phase (blue box) and an exponential growth phase (red box and red line). (B and C) *r*_*Glu*_ and *r*_*Gly*_ showing distinct metabolic profiles during the lag phase and the exponential growth phase. The REGP method is used to calculate the exchange fluxes during the exponential phase (solid red line). Exchange fluxes in the lag phase are estimated by connecting a straight line from the fresh media sample at t = 0 h to the projected exo-metabolite concentration at t = 15 h based on REGP calculations (blue dashed line). See also Table S1.

We measured the exo-metabolite concentrations of exponentially growing MCF10A cells at five time points during the exponential growth steady state (see Table S1) and used both the CORE (Figure 1A) and REGP (Figure 1B) methods to calculate the exchange fluxes of exo-metabolites, *r*_*m*_. Figure 1C shows the comparison between *r*_*m*,*REGP*_ and *r*_*m*,*CORE*_ in the MCF10A cell line, as well as the *r*_*m*,*CORE*_ of 11 cell lines of the NCI-60 panel that were previously considered reliable.^3^^,^^4^^,^^20^ By convention, consumption of metabolites (e.g., glucose) is represented by a negative flux and release of metabolites (e.g., lactate) by a positive flux. As expected, *r*_*m*,*CORE*_ were comparable between MCF10A cells and the 11 cell lines of the NCI-60 panel (Figure 1C). However, the *r*_*m*,*REGP*_ and *r*_*m*,*CORE*_ in MCF10A cells, based on the same raw metabolite measurements and cell count data, were substantially different. For several exo-metabolites, for example glutamate and glycine, *r*_*m*,*CORE*_ values were positive, indicating that cells were releasing these metabolites into the culture media, while *r*_*m*,*REGP*_ values were negative, indicating that cells were consuming these metabolites as nutrients. As the CORE method encompasses both the lag phase and exponential growth phase (Figure 1A), whereas the REGP method calculates the exchange flux during exponential growth only (Figure 1B), this reflects that cell metabolism differs between the lag phase and exponential growth (Figure 2). Our results indicate that glutamate is released by the cells during lag phase and consumed during exponential growth (Figure 2B). Similarly, consumption of glycine differs between lag phase and exponential growth (Figure 2C).

For other nutrients, such as glucose, glutamine, and other amino acids, in general, we observed that *r*_*m*,*REGP*_ for nutrient consumption was larger (that is, more negative) than *r*_*m*,*CORE*_, consistent with a higher nutrient consumption rate during exponential growth compared to the lag phase (Figure 1C). For lactate, we observed that the exchange flux for lactate release was smaller (that is, less positive) by the REGP method (Figure 1C), suggesting that lactate production is elevated during the lag phase and reduced during exponential growth.

### Simulating steady-state cell growth

**Figure 3:**
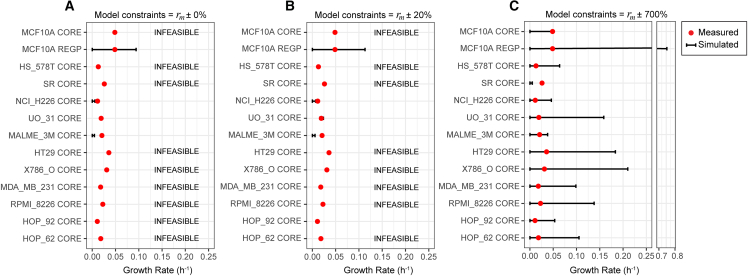
FVA simulation of cell growth rates Each cell line-specific GEM was constrained with the corresponding cell line-specific exo-metabolite exchange fluxes (*r*_*m*_) with a flexibilization factor of 0% (A), 20% (B), and 700% (C). Rows labeled as infeasible indicate that no solution exists that satisfies all constraints. Black bars, FVA-simulated minimum and maximum *in silico* cell growth rate for each cell line. Red dots, experimentally measured growth rate for each cell line. In none of the simulations did the minimum and maximum biomass flux equal zero (max = min = 0). See also Table S2.

A common way to benchmark FBA- and FVA-based GEM simulations is to estimate the cell growth rate,^3^^,^^21^ which can be easily validated experimentally. To do this, we first constructed cell line-specific GEMs by tINIT^22^ using cell line-specific transcriptomics data, generated in-house for MCF10A cells (Table S2; GEO accession GSE293588) and mined from the Cancer Cell Line Encyclopedia^23^ for the 11 cell lines of the NCI-60 panel. We then specified the metabolites that are present in the cell culture media (Ham’s medium), followed by constraining the exo-metabolite exchange fluxes (Figure 1C). For MCF10As, either *r*_*m*,*CORE*_ or *r*_*m*,*REGP*_ was used; for the 11 cell lines of the NCI-60 panel, only the available *r*_*m*,*CORE*_ were used (see Figure 3), which were mined from a previously described dataset,^3^^,^^4^ accessible in Robinson et al.^24^ Based on these input data as model constraints, the range of feasible *in silico* growth rates were simulated by maximizing and minimizing biomass production. We found that the *r*_*m*,*CORE*_-constrained MCF10A model was infeasible (Figure 3A), meaning that the *in silico* cell was unable to “grow” with the CORE-calculated metabolite uptake and secretion rates. In contrast, the *r*_*m*,*REGP*_-constrained MCF10A model was feasible (Figure 3A). Critically, the experimentally measured growth rate fell within the GEM-simulated solution space (Figure 3A), indicating that GEM simulations are physiologically relevant when using *r*_*m*,*REGP*_ as constraints, but not with *r*_*m*,*CORE*_. Similar to the *r*_*m*,*CORE*_-constrained MCF10A model, most of the *r*_*m*,*CORE*_-constrained models of the NCI-60 panel cell lines were also either infeasible or do not contain the experimentally measured growth rate within the simulated solution space (Figure 3A).

As a sensitivity analysis, we included a flexibilization factor ranging from 0% to 20% for all *r*_*m*_ used as model constraints (i.e., lb = 0.8∗*r*_*m*_; ub = 1.2∗*r*_*m*_) and found that that this did not play a role in determining model feasibility or the physiological relevance of the simulations (Figures 3A and 3B). The *r*_*m*,*CORE*_-constrained MCF10A model only produced physiologically relevant simulations when the flexibilization factor was increased to 700%; even at this point, the *r*_*m*,*CORE*_-constrained model of the SR cell line still performed poorly (Figure 3C).

### Organization of the feasible flux space

**Figure 4:**
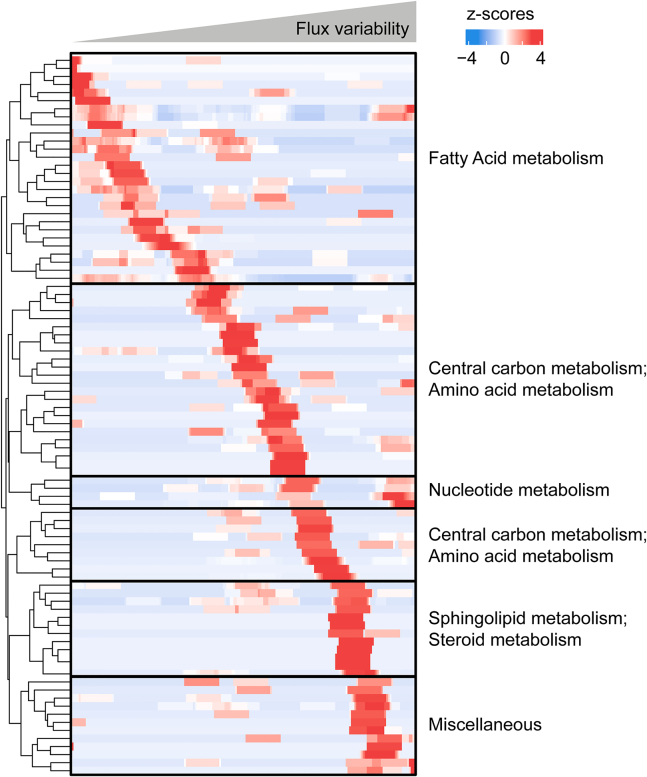
Organization of the feasible flux space of MCF10A cells The MCF10A-specific GEM was constrained with *r*_*m*,*REGP*_ and the measured growth rate, both with a flexibilization factor of 20%. FVA was performed to determine the flux variability (i.e., the feasible solution space) for every metabolic reaction. The fraction of each metabolic subsystem was calculated in a sliding window of 200 reactions of increasing flux variability, followed by a *Z* score transformation to facilitate comparison. For ease of visualization, *Z* scores of >3 or < −3 were set to 3 or −3, respectively. Rows represent metabolic subsystems, and columns represent a window. Metabolic subsystems were grouped into larger metabolic pathway categories to facilitate visualization and interpretation; the raw *Z* scores with corresponding metabolic pathways can be found in Table S4. See also Tables S3 and S4.

We then took the *r*_*m*,*REGP*_-constrained MCF10A model as described above and added a constraint of the biomass production reaction with the experimentally measured growth rate, to produce a constrained GEM of the MCF10A cell line that makes use of all available data. We used this model to explore the feasible flux space of the entire metabolic network of the cell in order to identify to what degree metabolic subsystems are constrained. Following FVA for every metabolic reaction, reactions were ranked by increasing flux variability (Table S3). A sliding window of 200 reactions with a step size of 10 was applied (the first window included reactions 1–200 and the second window, reactions 11–210). For each window, we calculated the fraction of reactions belonging to each metabolic subsystem (subsystems as defined by Human-GEM). For each subsystem, *Z* scores were calculated based on the mean fraction and standard deviation across all windows. The representation of metabolic subsystems across the flux variability spectrum is visualized in Figure 4 and listed in Table S4, where each row represents a subsystem and each column a window. This analysis showed that metabolic subsystems related to fatty acid metabolism, including fatty acid biosynthesis pathways and beta oxidation pathways, exhibited low variability in reaction flux (Figure 4). In contrast, amino acid metabolism (AAM) and most central carbon metabolism (CCM) pathways showed intermediate to high levels of variability (Figure 4), even though the exo-metabolite exchange fluxes used as model constraints were all related to CCM (glucose, lactate) and AAM, consistent with previous observations.^14^^,^^25^ For nucleotide metabolism, we observed two distinct regions in this analysis, one with intermediate variability and another with high variability. Finally, we found that reactions in sphingolipid and steroid metabolism, as well as miscellaneous reactions such as pool reactions and artificial reactions necessary for model simulations, exhibited high flux variability (Figure 4).

## Discussion

Understanding human cell metabolism at the systems level is of critical interest in many areas of health and medicine. GEM-based simulations have shown very promising applications in microorganisms,^9^^,^^10^^,^^11^ but obtaining the necessary input data in human cells has proven to be difficult. A number of methodologies have been developed to leverage transcriptomics data as model constraints,^26^^,^^27^^,^^28^^,^^29^^,^^30^ with mixed results,^31^ likely because metabolic fluxes are poorly reflected by the abundance of (transcripts of) enzymes in a cell. More recently, exo-metabolite exchange fluxes have been used,^3^^,^^9^^,^^14^^,^^32^ based on a comparison of exo-metabolite concentrations between the “spent” and “fresh” media.^4^^,^^14^ The caveat of this method is that it violates the steady-state assumption of FBA and FVA and thus should be used with caution. To address this limitation, we applied a multiple-time point regression approach, which collects exo-metabolite fluxes exclusively during the exponential growth phase (Figures 1B and 1C). Our results indicated a substantial difference in exchange fluxes in different phases of cell growth (Figure 2), underscoring the importance of distinguishing between growth phases when studying cell metabolism. With the exponential growth-phase exchange fluxes as model constraints, GEM simulations were biologically plausible, with the measured cell growth rate falling within the simulated solution space (Figure 3). This allowed us to explore the entire metabolic network of the cell with a physiologically relevant flux profile, revealing a distinct organization of the feasible flux space by metabolic subsystems (Figure 4).

Previously, cell-specific GEMs constrained by the exchange fluxes of glucose, lactate, and threonine (all calculated by the CORE method) were shown to predict the cell growth rate to a reasonable degree of agreement with experimentally measured cell growth rates.^3^ However, incorporation of additional (CORE-calculated) exo-metabolite exchange fluxes, with a flexibilization factor of up to 20%, led to a large number of infeasible models (Figures 3A and 3B), suggesting underlying problems with the model constraints and the biological relevance of the simulations. With the REGP method, model simulations remained feasible with a larger number of measured exo-metabolite exchange fluxes, and the feasible flux space was constrained to a biologically plausible region (Figure 3). We found that the maximum *in silico* growth rate exceeded the experimentally determined growth rate by approximately 2-fold (Figures 3A and 3B). This suggests that a portion of the consumed nutrients is diverted into non-growth-related metabolic tasks, consistent with the notion that, perhaps with the exception of fast-growth cancer cells, human cells do not operate to solely maximize growth.^31^^,^^33^

Our results show that even though the input constraints of our model were all related to CCM (glucose, lactate) or AAM (see Figure 1C), there is, nevertheless, an intermediate level of flux variability in these subsystems (Figure 4). This is in line with previous work showing that these subsystems do not operate at full capacity in growing cells.^25^^,^^34^ In contrast, we found that reactions in fatty acid metabolism exhibited low flux variability, while reactions in sphingolipid metabolism and steroid metabolism exhibited high flux variability, likely reflecting the degrees of connectivity (i.e., pathway branching) in these subsystems.^3^^,^^35^

Even though regression-based methods for determining exchange fluxes have been established in other systems,^15^^,^^16^^,^^17^^,^^18^^,^^19^ our study applies and validates this approach in a human cell line, showing it that substantially enhances the biological accuracy of GEM simulations compared to the conventional CORE method (Figure 3). To further confirm the robustness of this method in human cell lines, future work should validate the approach across additional human cell lines and conditions. While this approach demands more resources for exo-metabolite measurements, we believe that it is crucial to profile the metabolic fluxes of human cells at the genome scale, which can lead to a better understanding of the metabolic process in healthy cells and the identification of potential metabolic targets in diseases.

### Limitations of the study

The REGP method has a higher demand for experimental resources compared to the conventional CORE method, as several exo-metabolite measurements (rather than only one) must be taken, at the appropriate time points, which must be first determined experimentally. Connected to this limitation, this study shows the application of the REGP method in only one human cell line, MCF10A. Application of our method across additional human cell lines and conditions is warranted in future studies.

## Resource availability

### Lead contact

Further information and requests for resources may be directed to and will be fulfilled by the lead contact, Rosemary Yu (r.yu@science.ru.nl).

### Materials availability

This study did not generate new unique reagents.

### Data and code availability

• Raw RNA-seq data are available at GEO, accession GSE293588.
• All other data and code used in this paper are available in the GitHub repository (https://github.com/Radboud-YuLab/FluxProfilingREGP). An archival DOI is provided in the key resources table.
• Any additional information required to re-analyze the data reported in this paper is available from the lead contact upon request.

## Acknowledgments

The Yu lab is supported by grants from the 10.13039/501100004622Dutch Cancer Society and the Radboud-Western Collaboration Fund. We thank Niky Thijssen for technical support during the experimental work.

## Author contributions

Conceptualization, R.Y.; data curation, C.A.M.H. and X.J.; formal analysis, C.A.M.H. and X.J.; funding acquisition, Y.C. and R.Y.; supervision, Y.C. and R.Y.; writing – original draft, C.A.M.H.; writing – review and editing, X.J., Y.C., and R.Y.

## Declaration of interests

The authors declare no competing interests.

## STAR★Methods

### Key resources table

**Table:** 

REAGENT or RESOURCE	SOURCE	IDENTIFIER
**Chemicals, peptides, and recombinant proteins**		

DMEM/F12	Gibco	11320033
MEGM Mammary Epithelial Cell Growth Medium SingleQuots	Lonza	CC-4136
Cholera toxin	Enzo Life Sciences	BML-G117
Penicillin-streptomycin	Gibco	15140
Trypsin-EDTA	Gibco	25300054
Trypan blue	Gibco	15250061

**Critical commercial assays**		

CyQUANT Cell Proliferation Assay	ThermoFisher Scientific	C7026
aTRAQ Kit	AB Sciex	4442673
KAPA RNA HyperPrep	Kapa Biosystems	KK8502
Quick-RNA Microprep	Zymo Research	R1051
Qubit HS	Invitrogen	Q32854
High Sensitivity DNA analysis	Agilent	5067–4626

**Deposited data**		

Data & code repository	This paper	doi:10.6084/m9.figshare.30784106
MCF10A RNA-seq	This paper	NCBI GEO accession number: GSE293588

**Experimental models: Cell lines**		

MCF10A	ATCC	Cat# CRL-10317; RRID: CVCL_0598

**Software and algorithms**		

MultiQuant	SCIEX	3.0.3
Seq2science	van der Sande et al.^36^	https://github.com/vanheeringen-lab/seq2science
Human1	Robinson et al.^3^^,^^24^	https://github.com/SysBioChalmers/Human-GEM/tree/v1.19.0
tINIT	Agren et al.^22^	https://github.com/SysBioChalmers/Human-GEM/tree/v1.19.0
MEMOTE	Lieven et al.^37^	https://github.com/opencobra/memote
MATLAB	MathWorks, Inc.	2023a
Gurobi solver	–	https://www.gurobi.com/
R	–	https://www.r-project.org/
COBRA toolbox	Heirendt et al.^38^	https://github.com/opencobra/cobratoolbox
NCI-60 metabolomics	Jain et al.^4^, Robinson et al.^3^^,^^24^	N/A

**Other**		

HPLC	Shimadzu	N/A
Nexera UHPLC system	Shimadzu	N/A
Qtrap 6500+ system	AB Sciex	1250140
BEH C18 column	Waters	186002353
NextSeq 2000 system	Illumina	N/A

### Experimental model and study participant details

#### Cell lines

MCF10A cells were purchased from ATCC (Cat# CRL-10317). Cells were cultured in DMEM/F12 (Cat# 11320033, Gibco) supplemented with MEGM Mammary Epithelial Cell Growth Medium SingleQuots Kit (Cat# CC-4136, Lonza) without GA-1000, along with 0.1 μg/mL cholera toxin (Cat# BML-G117, Enzo Life Sciences) and 100 U/mL penicillin-streptomycin (Cat# 15140, Gibco). Cells were tested for mycoplasma contamination routinely.

### Method details

#### Cell proliferation assays

Absolute cell counts were measured at 22, 26, 30, 46, 50, and 54 h after seeding using the CyQUANT Cell Proliferation Assay kit (Cat# C7026, ThermoFisher Scientific) with a CLARIOstar Plus plate reader (BMG LABTECH). Cell proliferation at all other time points were measured by Incucyte ZOOM (Essen Bioscience), then converted to absolute cell counts based on the corresponding cell counts from the CyQUANT measurements.

#### Biomass determination

Cells were harvested with 0.05% trypsin-EDTA (Cat# 25300054, Gibco) and counted using 0.4% trypan blue (Cat# 15250061, Gibco) in a TC20 Automated Cell Counter (BioRad). The cell suspension was transferred into pre-weighed Eppendorf tubes and pelleted by centrifugation at 200*g* for 5 min. Pellets were dried in a microwave at 360 W for 20 min, and desiccated in a desiccator for >3 days.

#### Exo-metabolite measurements

Sampling for exo-metabolites was done during cellular exponential growth phase between 22 and 30 h after seeding by collection of culture supernatant. Glucose and lactate concentrations were quantified as described before,^39^ using an HPLC (Shimadzu) with an Aminex HPX-87H column (Cat# 1250140, BioRad) at 65°C and an IR detector. The column was eluted with 5 mM H_2_SO_4_ at a flow rate of 0.6 mL/min for 26 min. Amino acids were quantified as described before,^40^ with the aTRAQ Kit (Cat# 4442673, AB Sciex) using a Nexera UHPLC system (Shimadzu) coupled to a Qtrap 6500+ system (AB Sciex) with a BEH C18 column (150 × 2.1 mm, 1.7 μm) (Cat# 186002353, Waters) at 50°C. A gradient elution of water (eluent A) and methanol (eluent B), both containing 0.1% formic acid and 0.01% heptafluorobutyric acid, were used as the mobile phases with a constant flow of 1 mL/min. The following MS parameters were used: Curtain Gas: 50; Collision Gas: Medium; IonSpray Voltage: 5500 V; Temperature: 500°C; Ion Source Gas 1: 60; Ion Source Gas 2: 50. The gradient method was: 2% B from 0 to 2.5 min, 2%–40% B from 2.5 to 3.9 min, held at 40% B until 4.2 min, 40%–90% B from 4.2 to 6.0 min, held at 90% B until 6.1 min, 90%–2% B from 6.1 to 8.0 min. Data acquisition and processing were performed using Analyst and MultiQuant 3.0.3 software. Following data QC, exo-metabolite exchange fluxes (*r*_*m*_) were calculated by CORE^4^ or REGP (see below).

#### Exo-metabolite exchange flux (*r*_*m*_) calculation by REGP

Exo-metabolite exchange fluxes (*r*_*m*_) were determined by calculating the ratio between spent media and unused media samples, then normalizing to the known metabolite concentrations of the cell culture media (DMEM/F12). Exo-metabolite concentrations were normalized for culture volume and cell dry weight, and a linear model was fitted to regress the concentrations against time. *r*_*m*,*REGP*_ is then taken as the slope of the linear model, i.e., the derivative of the fitted metabolite concentration $\left[\hat{m}\right]$ with respect to time *t* (Figure 1B). Goodness of fit was determined by R^2^, with an arbitrary cutoff of 0.7.

#### RNA sequencing

RNA was sampled at 27 h after seeding. RNA was extracted using the Quick-RNA Microprep Kit (Cat# R1051, Zymo Research) according to the manufacturer’s protocol. Libraries were prepared from 300 ng RNA with the KAPA RNA HyperPrep Kit with RiboErase (HMR) (Cat# KK8502, Kapa Biosystems). RNA fragmentation was performed for a desired library insert size of 200–300 bp by fragmentation for 6 min at 94°C. Library concentrations were determined using the Qubit HS kit (Cat# Q32854, Invitrogen). Library size distribution was determined using a High Sensititivy DNA analysis (Cat# 5067-4626, Agilent) on a Bioanalyzer 2100 (Agilent). Libraries with an average between 300 and 400 bp were loaded on the NextSeq 2000 system (Illumina). Reads were quality controlled, mapped to the human genome hg38, and counted by Seq2science,^36^ available at https://github.com/vanheeringen-lab/seq2science.

#### Genome-scale metabolic modeling

The consensus Human-GEM, Human1 v1.12.0,^3^ was used for all procedures detailed below. Human-GEM is a ‘generic’ model which contains all observed metabolites and reactions in human cells. For each cell line, contextualized models were constructed using tINIT,^22^ where the generic Human-GEM is pruned in a cell line-specific manner based on whether or not the (transcript of) an enzyme is expressed. MEMOTE was applied to test model validity, which confirmed that all models had a consistency score >85%.^37^ We used transcriptomics data measured in-house (MCF10As) or mined from CCLE,^23^ with an arbitrary expression level cutoff of 1 TPM. Each cell line-specific model was first constrained with components of the growth media (without specifying the exchange fluxes), and model feasibility was verified under these unconstrained conditions. The models were then further constrained by the measured exchange fluxes with a flexibilization factor ranging from 0% to 700%. For simulation of growth rate, the range of feasible *in silico* growth rate was determined using FVA by sequentially minimizing and maximizing the biomass reaction, as implemented in the COBRA toolbox.^38^ The MCF10A-specific model was further constrained with the measured growth rate to perform FVA for all reactions. The flux variability for each reaction, i.e., max(flux) – min(flux), is sorted from lowest to highest. A sliding window of 200 reactions with a step size of 10 reactions across the sorted list was used to assess the variation in reaction flux across different subsystems. Within each window, Z-scores were calculated for the fraction of reactions per subsystem in a given window by comparing the fraction to the overall mean and standard deviation across all windows. Exchange reactions, transport reactions, and subsystems with less than 5 reactions, were excluded from the visualization of this analysis in Figure 4. All simulations were performed using MATLAB 2023a (MathWorks, Inc.) with Gurobi solver v10.0.1 (Gurobi Optimizer).

### Quantification and statistical analysis

Procedures related to metabolic modeling are described in detail in the sections above and were implemented in MATLAB (2023a). Numerical analyses and graphics are done in R v4.2.3.

## Supplemental information

Table S1. Exo-metabolite concentrations of MCF10A cells, related to Figures 1 and 2

Table S2. TPM data of MCF10A cells, related to Figure 3

Table S3. FVA results, related to Figure 4

Table S4. Raw *Z* scores per metabolic subsystem, related to Figure 4
